# Identification of Genetic Variants in 65 Obesity Related Genes in a Cohort of Portuguese Obese Individuals

**DOI:** 10.3390/genes12040603

**Published:** 2021-04-19

**Authors:** Catarina Ginete, Bernardo Serrasqueiro, José Silva-Nunes, Luísa Veiga, Miguel Brito

**Affiliations:** 1 H&TRC Health and Technology Research Center, Escola Superior de Tecnologia da Saúde de Lisboa, Instituto Politécnico de Lisboa, 1990-096 Lisbon, Portugal; catarina.ginete@estesl.ipl.pt (C.G.); bernardo.serrasqueiro@gmail.com (B.S.); silvanunes2004@yahoo.com (J.S.-N.); luisa.veiga@estesl.ipl.pt (L.V.); 2 Department of Endocrinology, Diabetes and Metabolism, Centro Hospitalar Universitário de Lisboa Central, 1150-199 Lisbon, Portugal; 3 NOVA Medical School/Faculdade de Ciências Médicas, New University of Lisbon, 1169-056 Lisbon, Portugal

**Keywords:** obesity, monogenic obesity, next-generation sequencing (NGS), obese women

## Abstract

Obesity is a major public health problem, which has a strong genetic component that interplays with environmental factors. Several genes are known to be implicated in the regulation of body weight. The identification of alleles that can be associated with obesity is a key element to control this pandemic. On the basis of a Portuguese population, 65 obesity-related genes are sequenced using Next-Generation Sequencing (NGS) in 72 individuals with obesity, in order to identify variants associated with monogenic obesity and potential risk factors. A total of 429 variants are identified, 129 of which had already been associated with the phenotype. Comparing our results with the European and Global frequencies, from 1000 Genomes project, 23 potential risk variants are identified. Six new variants are discovered in heterozygous carriers: four missense (genes ALMS1-NM_015120.4:c.5552C>T; SORCS1-NM_001013031.2:c.1072A>G and NM_001013031.2: c.2491A>C; TMEM67-NM_153704.5:c.158A>G) and two synonymous (genes BBS1-NM_024649.4:c.1437C>T; TMEM67-NM_153704.5:c.2583T>C). Functional studies should be performed to validate these new findings and evaluate their penetrance and pathogenicity. Regardless of no cases of monogenic obesity being identified, this kind of investigational study is important when we are still trying to understand the aetiology and pathophysiology of obesity. This will allow the identification of rare variants associated with obesity and the study of their prevalence in specific populational groups.

## 1. Introduction

Obesity is a major public health problem. The World Health Organization estimated that in 2016 more than 650 million adults (13% of the world’s population) would be obese and 1.9 billion (39% of the world’s population) would be overweight [1]. In Portugal, it was estimated that in 2017, 22.3% of the population was obese [2].

Obesity is a complex disease influenced by the interaction of genetic and environmental factors [3] and a major risk factor for the development of other conditions, such as type 2 diabetes mellitus, hypertension, cardiovascular diseases and even certain types of cancer. The presence of these severe co-morbidities is responsible for the increased risk of mortality in these individuals [4,5].

Obesity can also be one of the phenotypic characteristics in syndromic diseases, such as the Prader-Willi syndrome, Bardet-Biedl syndrome or Albright’s hereditary osteodystrophy, but this only accounts for a small percentage of obesity cases.

Moreover, cases of non-syndromic monogenic obesity are also very rare, accounting for less than 5% of diagnoses in Europe [6]. Non-syndromic monogenic obesity may have a dominant or recessive inheritance, resulting from a pathogenic mutation in genes with an essential role in maintaining energy homeostasis by participating in the leptin-melanocortin signalling pathway. These include the LEP, LEPR, MC4R, POMC and PCSK1 genes. A mutation with loss of function in one of these genes in homozygous carriers will cause disruption in this signalling pathway, resulting in monogenic obesity [3,4,6]. Approximately 140 variants in these genes associated with non-syndromic monogenic obesity are documented in the ClinVar (https://www.ncbi.nlm.nih.gov/ClinVar (accessed on 3 January 2020)) and Ensembl (https://www.ensembl.org (accessed on 3 January 2020)) databases.

Most often, obesity has a polygenic and multifactorial origin. Polygenic, since it results from the presence of several common variants. When these variants are isolated, they have no or little metabolic effects, but when they are together, they increase the susceptibility to gain weight [7]. Multifactorial, since it is related not only to predisposing genetic factors, but also to the “obesogenic” environment, associated with a sedentary lifestyle (imbalance between intake and energy expenditure) and social and cultural factors. These non-genetic factors, such as nutrition and physical activity, also seem to have an impact on the modulation of gene activity, due to changes in the epigenetic signatures [3].

Therefore, the identification of variants associated with obesity is extremely relevant for differential diagnosis, for treatment selection in these patients and for prenatal diagnosis in families where a known variant was found.

Despite all the research studies already conducted, the aetiology of obesity is still far from being fully understood. The development of further studies, in order to identify new genes and variants involved in the regulation of body weight and obesity, and the study of their prevalence in a specific population, will allow us to move towards a future focused on the patient, with more effective prevention, follow-up and personalised treatment.

With this in mind, the primary aim of this research is to find new genetic variants linked to obesity and assess its prevalence in a Portuguese population.

## 2. Materials and Methods

We considered 65 genes (exons and exon-intron junctions), previously identified as risk factors for obesity development, which were sequenced by NGS, in a Portuguese group of obese/overweight individuals.

### 2.1. Patient Selection

The study sample consisted of 72 individuals (55 females, aged between 21 and 67 years and with a body mass index (BMI) between 26.9 and 68.0 kg/m^2^, followed up at the Multidisciplinary Obesity Department of the Centro Hospitalar Universitário de Lisboa Central (Curry Cabral). The collection of the samples occurred after the individual signature of an informed consent. The protocol was approved by the Ethical Committee of ESTeSL (CE-ESTESL-No.41-2018).

### 2.2. Laboratory Analysis

Oral epithelium cells were collected with swab and DNA was extracted with ExtractMe kit^®^ from BLIRT SA. The success of the extraction was evaluated by a fluorimetric method using the QuBit^®^ equipment and the Qubit dsDNA high-sensitivity assay, from ThermoFisher Scientific (target-selective dyes bound to DNA and emit fluorescence). For all samples, some after 2 elutions, it was possible to reach the minimum concentration required for subsequent sequencing of the panel of 65 genes by NGS.

After obtaining the desired DNA concentrations, the libraries were prepared with TruSight One kit^®^ from Illumina, Inc. (San Diego, CA, USA) respecting the manufacturer’s instructions. Sequencing was performed on the NextSeq550 equipment from Illumina, Inc using the NextSeq 500/550 High Output Kit v2 (150 cycles).

The gene panel used includes genes associated with monogenic obesity and the leptin-melanocortin signalling pathway (LEP, LEPR, PCSK1, POMC and MC4R) [3], genes associated with insulin signalling pathway (IRS1, IRS2, IRS4 and SORCS1) [8,9,10], genes associated with lipid metabolism (ADRB1 and ADRB3, PRKAR1, PTEN and SPG11), [11,12,13,14] other genes associated with appetite regulation (BDNF, NTRK2, LRP2, MCHR1 and SLC6A14 [13,14,15,16,17] genes associated with syndromic obesity (ALMS1 and BBS family), among other genes previously associated with obesity (Table 1) [3,17,18,19].

### 2.3. Data Analysis

The analysis of the obtained data began with the pre-processing of the sequences, and the variant calling, in the Enrichment and BWA Enrichment applications of Illumina, Inc. For the variant annotation, the software Illumina Variant Studio 3.0 was used. Only variants detected in both applications (Enrichment and BWA Enrichment of Illumina, Inc.) and passing the PASS filter of Illumina Variant Studio 3.0, were considered for the continuation of the study. Variants outside exons and exon-intron regions (up to 12 bases) were also excluded.

To check the quality of readings, coverage and read depth, Software IGV-Integrative Genomics Viewer was used.

For the analysis of the identified variants, prediction of their impact and clinical relevance, we used ClinVar (NCBI), a public database, where associations between human genetic variants and phenotypes, scientifically based, are reported, and PolyPhen-2, a software available online, which predicts the possible impact of amino acid substitutions on the stability and function of human proteins. In this software, SNPs are functionally annotated, coding SNPs are mapped to gene transcripts, protein sequence annotations and structural attributes are extracted and conservation profiles are developed. The probability of the missense mutation being harmful is then calculated using a combination of these properties. In Polyphen, we can find two variant classifiers, HumDIV and HumVAR. Only predictions from HumDIV will be presented in this study, since is considered preferential for rare alleles [20]. We also resort to the Human Splicing Finder to evaluate the impact on splicing, of intron, missense and synonymous variants located in splicing regions (1–3 bases of the exon, 1–8 bases of the intron).

To identify risk variants associated with obesity, the frequencies of the variants identified in this study were compared with European and Global frequencies in the 1000 Genomes project (https://www.internationalgenome.org (accessed on 6 January 2020) and, when not reported, in TopMed (https://www.nhlbiwgs.org/ (accessed on 6 January 2020). To be considered a risk allele, the difference between the frequency in the studied sample and the European and Global reference frequency had to be equal to or greater than 1.41% (corresponding to the proportion of two alleles in the calculated sample frequency−2/(2 × 71)).

## 3. Results

Of the 72 samples analysed, a coverage smaller than 80% was obtained in eight samples. After applying the PASS filter Illumina Variant Studio, variants were detected in all samples, except for one. The use of a non-invasive method for sample collection (oral epithelium cells collected with swabs) was probably the reason for the low coverage detected in these samples. To perform the technique, it was necessary to obtain a DNA concentration higher than 5 ng/μL per sample, which for some samples was only possible after a second extraction and elution. Effectively, the efficiency of DNA recovery and extraction from swabs is usually, regardless of the material, less than 50% [21].

Globally, 429 different variants were identified in 63 of the 65 genes under study: 423 snv’s (single nucleotide variant), 1 mnv (multiple nucleotide variant), 2 insertions and 3 deletions. In absolute numbers, 4348 variants were detected: 4318 snv’s, 6 mnv’s, 21 insertions and 3 deletions.

No variants were detected in PHF6 and PTEN genes. After verifying the mean coverage value of these sequences, we could rule out the suspicion of inefficacy of the enrichment probes.

### 3.1. Variant Consequences

Analysing the 429 different variants identified (Table 2), missense variants were the most frequent, counting 48.25%, followed by the synonymous variants with 41.49%. According to the 1000 Genomes Project, 52.09% of these variants have a European prevalence of less than 1%.

According to PolyPhen HumDIV, of the 207 missense variants identified, 125 are predicted to be benign, 35 are possibly damaging and 44 probably damaging. Three variants have unknown impact. In absolute numbers, 1701 missense variants were detected, 1435 benign, 117 possibly damaging and 145 probably damaging.

Table 3 describes the clinical associations that may be related to obesity, the number of variants identified and the number of samples where these variants were detected.

A total of 129 variants are potentially associated with obesity, according to ClinVar and the consulted bibliography, some of which have more than a clinical association. Of these variants, seven are classified as a risk factor, one is classified as probably pathogenic and three as pathogenic (Table 4).

### 3.2. Variant Incidence

**Figure 1:**
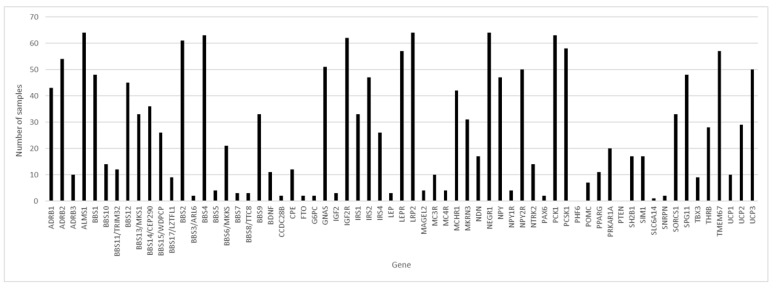
Number of samples where variants were detected, by gene. The Y axis represents the number of samples, and the X axis the name of genes.

Of the 65 genes studied and considering the 64 samples with coverage greater than 80.00%, in three genes, variants were found in 100% of the samples: ALMS1, LRP2 and NEGR1 (Figure 1), followed by the BBS4 and PCK1 genes, with variants in 98.44% of the samples.

**Figure 2:**
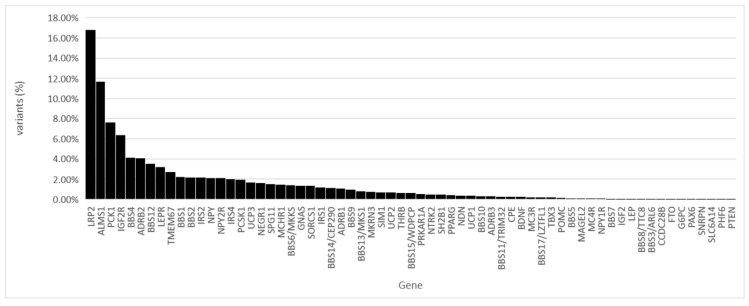
Percentage of samples where variants were detected, by gene. The Y axis represents the percentage of samples with variability, and the X axis the name of the genes.

Of all the identified variants, 16.80% were found in the LRP2 gene and 11.66% in the ALMS1 gene (Figure 2).

In the 64 samples, we found variants in an average of 26.69 genes and an average of 66.2 variants per sample.

### 3.3. Identification of Potential Risk Alleles

The identified potential risk alleles associated with obesity are described in Table 5.

Although they did not fully meet the criteria defined previously, variants rs183867145, rs147058423 and rs189273089 (found in LRP2 gene) were also considered as potential risk variants associated with obesity. Effectively, according to the 1000 Genomes Project, variant rs183867145 was only identified in one American heterozygous individual, variant rs147058423 in a European heterozygous individual and variant rs189273089 in two heterozygous carriers, one American and one African. The three variants were detected each in two individuals in our sample.

In five variants, the frequency of the minor allele in the sample was lower than the European and Global frequencies, according to 1000 Genomes project (Table 6). These findings suggest that the c.724-8G allele of the BBS1 gene, c.196G of the BDNF gene, c.2512A of the BBS14/CEP290 gene, c.745C of the IGF2R gene and c.84T of the SLC6A14 gene constitute potential risk alleles for obesity.

### 3.4. New Variants

In the variant analysis, six new variants were detected, all in heterozygous carriers. These variants, localised in genes ALMS1, BBS1, SORCS1 and TMEM67 were not referenced in the consulted databases. The variant c.5552C>T, in the ALMS1 gene, results in a missense variant, where a Proline is replaced by a Leucine in the amino acid 1851. This variant has a PolyPhen prediction of possibly damaging. The variant c.1437C>T, in the BBS1 gene, results in a synonymous variant. The variant c.1072A>G, in the SORCS1 gene, results in a missense variant, where an Arginine is replaced by a Glycine in the amino acid 358. This variant has a PolyPhen prediction of possibly damaging. The variant c.2491A>C, in the SORCS1 gene, results in a missense variant, where a Threonine is replaced by a Proline in the amino acid 831. This variant has a PolyPhen prediction of probably damaging. The variant c.158A>G, in gene TMEM67, results in a missense variant, where a Glutamine is replaced by an Arginine in the amino acid 53. This variant has a PolyPhen prediction of benign. The variant c.2583T>C, in gene TMEM67, results in a synonymous variant.

To validate these results, functional studies should be performed.

## 4. Discussion

In the 72 individuals studied, no cases of monogenic obesity were recognised. In all samples, several variants were identified that constitute risk factors associated with obesity (between 3 and 12 variants per individual), which may influence the development of this phenotype in these individuals.

Of all the variants identified in this study, only five (Table 7), found in heterozygous carriers, have a ClinVar classification of pathogenic or probably pathogenic.

The four syndromes referenced in Table 6 have autosomal recessive transmission, and, with the exception of the Donnai-Barrow Syndrome, obesity may be part of the phenotype [14,22,23]. The variant rs2229707 is described as associated with severe obesity and was detected in a study conducted by Argyropoulos et al., in an obese woman in heterozygosity (39 years), in three obese descendants in homozygosity (11, 14 and 20 years—BMI is higher in older individuals) and in a non-obese descendant (9 years) in heterozygosity [24]. The information from this study and the other consulted bibliography is not enough to define whether this mutation may be associated with monogenic obesity and whether it has autosomal dominant or recessive heredity. In one of our samples, this variant was detected along with another possibly damaging variant in the UCP3 gene (rs8179180), both in heterozygosity. This sample belongs to an obese woman, whose father and mother, three siblings and two children are obese. It would be interesting to carry out the same study, sequencing the 65 genes, to all family members (including non-obese family members, who are not included in the available family history). Thus, we could test the penetrance and evaluate the clinical significance, and association with obesity, of these two variants identified in the UCP3 gene.

### Limitations

One of the limitations of this study was the use of a sample collected by a non-invasive method (samples of oral epithelium cells collected by swab). This was probably the reason for the low coverage detected in some samples and the lack of results in one of the samples. The chosen NGS technique also failed to detect chromosomal structural changes such as massive deletions, insertions, inversions, or duplications, as well as epigenetic changes that may have occurred [9,25]. The lack of detailed clinical and family history of the individuals under study, namely associated co-morbidities, cases of childhood obesity or the identification of non-obese family members, and the non-sequencing of samples of relatives (ascending and descending), hindered the interpretation of the found variants, although it would help in the establishment of correlations between alleles and phenotypes. The variant frequency analysis was also hampered by the lack of a control group and the use of a database, the 1000 Genomes Project, in which obesity was not an exclusion criterion and there was no representation from Portugal [26].

## 5. Final Considerations

This study aimed to identify new genetic variants associated with obesity and to determine its prevalence in the Portuguese population. The development of similar studies may allow in the future to easily identify individuals at high risk of developing obesity in order to define more effective preventive strategies. In that sense, it will allow personalised medicine to be applied to the obesity field.

## Figures and Tables

**Table 1:** Gene panel (genes previously associated with obesity).

ADRB1	BBS8/TTC8	CPE	LRP2	NTRK2	SLC6A14
ADRB2	BBS9	FTO	MAGEL2	PAX6	SNRPN
ADRB3	BBS10	G6PC	MC3R	PCK1	SORCS1
ALMS1	BBS11/TRIM32	GNAS	MC4R	PCSK1	SPG11
BBS1	BBS12	IGF2	MCHR1	PHF6	TBX3
BBS2	BBS13/MKS1	IGF2R	MKRN3	POMC	THRB
BBS3/ARL6	BBS14/CEP290	IRS1	NDN	PPARG	TMEM67
BBS4	BBS15/WDPCP	IRS2	NEGR1	PRKAR1A	UCP1
BBS5	BBS17/LZTFL1	IRS4	NPY	PTEN	UCP2
BBS6/MKKS	BDNF	LEP	NPY1R	SH2B1	UCP3
BBS7	CCDC28B	LEPR	NPY2R	SIM1	

**Table 2:** Variants classified by consequence.

Variant Consequences	Number of Variants	Number of Variants with a European Prevalence <1% According to the 1000 Genomes Project
Missense variant	207	122
Synonymous variant	178	75
Intron variant	38	23
3′ prime UTR variant	3	0
Nonsense variant	2	2
Frameshift variant	1	1
Inframe deletion	1	1
Inframe insertion	1	0

**Table 3:** Clinical association of the identified variants potentially associated with obesity.

Clinical Associations	Number of Different Variants	Total Number of Variants	Number of Samples
Obesity Susceptibility	3	44	35
Obesity	5	40	34
Monogenic obesity	13	228	66
Syndromic obesity	94	671	71
Childhood obesity	2	13	13
Type 2 Diabetes Mellitus	5	45	32
Monogenic Diabetes	12	26	20
Insulin resistance	2	23	20
Influence on BMI	3	35	23
Leptin deficiency or disfunction	2	3	3
Leptin receptor deficiency	5	138	59
Metabolic syndrome susceptibility	2	77	50
Thyroid hormone resistance	3	27	27

**Table 4:** Clinical association of the identified variants potentially associated with obesity.

Gene	dbSNP ID	Ho	He	ClinVar	Disease	PolyPhen HumDIV
ADRB2	rs1042713	23	23	Risk factor	Metabolic syndrome	Benign
ADRB2	rs1042714	31	20	Risk factor	Metabolic syndrome, obesity	Benign
ADRB3	rs4994	0	10	Risk factor	Obesity	Benign
IRS1	rs1801278	1	11	Risk factor	Insulin resistance	Possibly damaging
IRS2	rs1805097	2	20	Risk factor	Diabetes Mellitus	-
PCSK1	rs6232	0	5	Risk factor	Obesity	Benign
POMC	rs28932472	0	2	Risk factor	Early-onset obesity	Probably damaging
BBS2	rs773417074	0	1	Prob pathogenic	Bardet-Biedl syndrome	-
MKKS	rs74315394	0	1	Pathogenic	Mckusick Kaufman syndrome	Probably damaging
SPG11	rs199588440	0	1	Pathogenic	Spastic paraplegia 11	
UCP3	rs2229707	0	1	Pathogenic	Severe obesity, T2 Diabetes	Benign

Ho—homozygotic; He—Heterozygotic.

**Table 5:** Potential risk alleles associated with obesity.

Gene	dbSNP ID	Variant		Freq (%)	Eur Freq (%)	Gl Freq (%)
ADRB2	rs1800888	c.491C>T	p.Thr164Ile	3.52	1.79	0.40
ALMS1	rs41291187	c.1868A>G	p.His623Arg	3.52	2.09	0.70
BBS9	rs11773504	c.1363G>A	p.Ala455Thr	21.83	19.58	17.13
IGF2R	rs76130099	c.5701G>A	p.Val1901Ile	2.11	0.60	0.42
IGF2R	rs2297367	c.6995+6C>T		10.56	4.87	7.09
IRS1	rs2234931	c.702G>A	c.702G>A(p. =)	11.27	8.55	5.29
LRP2	rs147058423	c.3110G>A	p.Arg1037Lys	1.41	0.10	0.02
LRP2	rs189273089	c.9592G>A	p.Glu3198Lys	1.41	0.00	0.04
LRP2	rs41268685	c.13250G>A	p.Gly4417Asp	2.82	1.19	0.58
LRP2	rs183867145	c.894A>G	c.894A>G(p. =)	1.41	0.00	0.02
LRP2	rs34104660	c.402C>A	c.402C>A(p. =)	9.86	8.15	4.75
LRP2	rs33954745	c.2376T>C	c.2376T>C(p. =)	9.86	8.45	7.63
NPY1R	rs5578	c.1121A>C	p.Lys374Thr	2.82	0.69	0.30
NTRK2	rs2289657	c.1848C>A	c.1848C>A(p. =)	8.45	4.37	5.81
POMC	rs80326661	c.641A>G	p.Glu214Gly	2.11	0.69	0.14
SPG11	rs80338869	c.7023C>T	c.7023C>T(p. =)	7.04	3.18	1.18
SPG11	rs79708848	c.1698T>G	p.Asp566Glu	2.82	1.39	0.52
TMEM67	rs117195541	c.2397T>C	c.2397T>C(p. =)	2.82	1.39	0.84

Freq—Frequency in the studied sample; Eur Freq—European frequency according to 1000 Genome Project; Gl Freq—Global Frequency according to the 1000 Genome Project.

**Table 6:** Variants in which the frequency of the minor allele is lower than European/global frequencies.

Gene	dbSNP ID	Variant		Freq (%)	Eur Freq (%)	Gl Freq (%)
BBS1	rs10896125	c.724-8G>C		17.90	23.96	24.26
BBS14/CEP290	rs11104738	c.2512A>G	p.Lys838Glu	2.86	4.87	9.70
BDNF	rs6265	c.196G>A	p.Val66Met	8.73	19.68	20.13
IGF2R	rs8191754	c.754C>G	p.Leu252Val	12.14	14.81	16.03
SLC6A14	rs12720074	c.84T>A	c.84T>A(p. =)	0.82	9.66	5.40

Freq—Frequency in the studied sample; Eur Freq.—European frequency according to 1000 Genome Project; Gl Freq.—Global Frequency according to the 1000 Genome Project.

**Table 7:** Pathogenic or probably pathogenic variants identified.

Gene	dbSNP ID	Variant	ClinVar	Disease	PolyPhen HumDIV
BBS2	rs773417074	c.627_628delTT p.Cys210SerfsTer20	Probably Pathogenic	Bardet-Biedl syndrome	-
LRP2	rs138269726	c.6160G>A p.Asp2054Asn	Pathogenic	Donnai Barrow syndrome	Probably damaging
MKKS	rs74315394	c.724G>T p.Ala242Ser	Pathogenic	Mckusick Kaufman syndrome	Probably damaging
SPG11	rs199588440	c.1951C>T p.Arg651Ter	Pathogenic	Spastic paraplegia 11	-
UCP3	rs2229707	c.304G>Ap.Val102Ile	Pathogenic	Severe obesity, T2 Diabetes	Benign

## Data Availability

The datasets used and analysed during the current study are available from the corresponding author on reasonable request.
